# Biologically effective dose (BED) escalation of stereotactic body radiotherapy (SBRT) for hepatocellular carcinoma patients (≤5 cm) with CyberKnife: protocol of study

**DOI:** 10.1186/s13014-020-1471-1

**Published:** 2020-01-28

**Authors:** Jing Sun, Aimin Zhang, Wengang Li, Quan Wang, Dong Li, Dan Zhang, Xuezhang Duan

**Affiliations:** Radiation Oncology Center, The Fifth Medical Center of PLA General Hospital (Beijing 302 Hospital), No. 100 Xi Si Huan Middle Road, Fengtai District, Beijing, 100039 China

**Keywords:** Biologically effective dose, Hepatocellular carcinoma, Stereotactic body radiotherapy, Protocol, CyberKnife

## Abstract

**Background:**

There is a lack of data on the biologically effective dose and the efficacy of stereotactic body radiotherapy in hepatocellular carcinoma patients, and this study was conducted to explore the relation between BED and efficacy.

**Methods:**

This study is designed as a mono-center study. The participants are randomized into three group, and received the following recommended schedule: 49Gy/7f, 54Gy/6f and 55Gy/5f with BED_10_ in correspondence to 83.3Gy, 102.6Gy and 115.5Gy. The primary outcome measures are to calculate local control rates (LC), overall survival rates (OS) and progression-free survival rates (PFS). The secondary outcome measures are to observe radiation-induced liver injury (RILD) rates, Child-Pugh score and indocyanine green retention rate at 15 min (ICG-R15) value before and after CK-SBRT. Moreover, gastrointestinal toxicities are also observed.

**Discussion:**

There is no uniform standard for CK-SBRT dose schedule of hepatocellular carcinoma. We propose to conduct a study determining the optimal CK-SBRT schedule of hepatocellular carcinoma patients (≤5 cm). The trial protocol has been approved by the Institutional Review Board of 302 Hospital of PLA (People’s Liberation Army). The Ethics number is 2017111D.

**Trail registration:**

Clinical trails number: NCT03295500. Date of registration: November, 2017.

## Background

There were 841,080 new liver carcinoma cases and 781,631 deaths worldwide in 2018 [1]. Stereotactic body radiotherapy (SBRT) was an option for hepatocellular carcinoma patients who are not suitable for radical surgery, such as liver transplantation, resection, radio-frequency ablation (RFA) [2]. CyberKnife SBRT (CK-SBRT) is a new type of stereotactic radiosurgery, which can be guided by respiratory synchronous tracking and fiducial markers tracking. With two tracking ways above, CK-SBRT has certain advantages in treating liver lesions that move constantly.

The overall survival rates of lung cancer and cervical cancer patients increased with the delivery of increasing biologically effective doses (BED) to lesions [3, 4]. However, there is a lack of data on the BEDs and the efficacy of SBRT in hepatocellular carcinoma patients. Moreover, it is also worth studying whether BED escalation can cause an increase in the incidence of radiation injury.

## Method and design

### Study design

This is a mono-center study to explore the efficacy and adverse reaction for hepatocellular carcinoma patients with BED_10_ escalation of CyberKnife SBRT (CK-SBRT).

### Primary outcome measures

1. Local control rate: LC is calculated starting from the date of CK-SBRT to progress (the lesion diameter is more than original tumor in contrast-enhanced CT or contrast enhanced MRI).
2. Overall survival rate: OS is defined starting from the date of CK-SBRT to the date of final follow-up or demise of patients.
3. Progression-free survival: PFS was estimated starting from the date of CK-SBRT to the date of disease progression or demise of patients.

### Secondary outcome measures

1. Radiation-induced liver injury (RILD) rate:

RILD included classic RILD and non-classic RILD. Classic RILD manifests as symptoms of fatigue, hepatomegaly, and anicteric ascites, etc. Additionally, the serum alkaline phosphatase level in these patients increases to more than twice the normal level, but the serum transaminase and bilirubin levels in remain normal [5, 6]. Non-classic RILD usually occurred in patients with hepatitis and cirrhosis who are also with markedly elevated serum transaminases (> 5 times the upper limit of normal) rather than elevated alkaline phosphatase or a decline in liver function (measured by a worsening of Child-Pugh score by 2 or more) [7].

RILD was evaluated every 3 days during CK-SBRT, every month for initial 3 months and every 3 months thereafter until 8 months after CK-SBRT.

2. Child-Pugh score was calculated every 3 days during CK-SBRT, every month for initial 3 months and every 3 months thereafter until 8 months after SBRT.
3. Indocyanine green retention rate at 15 min (ICG-R15) value was estimated every 3 days during CK-SBRT, every month for initial 3 months and every 3 months thereafter until 8 months after SBRT.
4. To observe acute and late gastrointestinal toxicities following CK-SBRT [8].

#### Inclusion criteria

The hepatocellular carcinoma patients were diagnosed by image examination and laboratory test or pathology.

1. The diameter of lesion ≤5 cm.
2. Age of 30–80 years old.
3. Child-pugh classification A or B.
4. ECOG PS score 0 or 1.
5. Leukocyte count≥2*10^9^/L, platelets count≥60*10^9^/L.
6. Kidney function is normal: Urea:2.9–8.2 mmol/L, Cr: 62-115umol/L.
7. Unsuitable to or rejecting other therapies, such as resection, liver transplantation, etc.
8. A life expectancy of ≥6 months.
9. All participants understand the research subject and sign a written informed consent document.

#### Exclusion criteria

1. With previous therapies, such as resection, liver transplantation, radiofrequency ablation, transarterial chemoembolization, etc.
2. The outline of lesion is not confirmed by image examination.
3. With hepatic or any other abdomen radiotherapy history before.
4. With severe internal medicine diseases.
5. Intractable ascites.
6. Distance between lesion and organs at risk (OARs: esophagus, stomach, intestine) is less than 5 mm.
7. The normal liver volume less than 700 cc.
8. With extrahepatic metastasis.
9. Pregnant women.
10. Participants who are in another trial while on study.

#### Radiation treatment planning

All participants are implanted with 4 to 6 fiducial markers 1 week prior to CT localization. The distance between markers and lesion is less than 6 cm. Before CT localization, a vacuum-bag is used for fixing the body, the arms and the legs (both arms are along the body, and both hands are on thighs). The acquired parameters of CT images are as follows: tilted angel of 0°; slice thickness of 1 mm; voltage of 120 KV tube current of 400 mA; pixel size of 512 × 512. When the patients received simulation, they need hold their breath with smooth breathing. We adopt contrast-enhanced CT or contrast-enhanced MRI as an auxiliary image for fusion. The radiation oncologists contour gross tumor volume (GTV), planning target volume (PTV) and organs at risk. GTV is defined as the visible lesion based on image examination. PTV expands 3-5 mm of GTV. The participants are randomized into three group, and received the following recommended schedule: 49Gy/7f, 54Gy/6f and 55Gy/5f with BED_10_ in correspondence to 83.3Gy, 102.6Gy and 115.5Gy.

All CK-SBRT plans are calculated by G4 CyberKnife MultiPlan (Version 4.0.2) and VSI CyberKnife MultiPlan (Version 4.6.1). The plans enclosed PTV with 75–90% isodose line of maximum dose equated to the prescribed dose. Normal tissues tolerance doses comply with AAPM TG-101 report [9].

#### Evaluation and follow-up

The CK-SBRT plan is delivered every day including on weekend. During CK-SBRT, the adverse reaction is evaluated every day. Physical examination and laboratory test are assessed every 3 days. In case patients present nausea and/or vomiting, they will receive corresponding drug treatment. The treatment schedule will be delayed when patients with uncontrollable vomiting and/or abnormal liver function. After CK-SBRT, the patients are followed up, which includes physical examination, laboratory test and image examination. The follow up period defines as every month for initial 3 months, and every 3 months thereafter until 2 years. Follow-up include The trail was planned to begin on September 2017.

#### A quality assurance

Three radiation oncologists and two physicists formed CyberKnife QA group.

#### Statistical analysis

LC, OS and PFS are estimated using the Kaplan-Meier method. LC, OS and PFS related group analyses of BED_10_ are performed using the log rank test. Univariate and multivariable hazard ratios are calculated using the Cox proportion hazard model. For comparisons between the baseline variables, the χ2 test and Fisher’s exact test were performed. *P* values< 0.05 are considered statistically significant.

## Discussion

There is no uniform standard for CK-SBRT dose schedule of hepatocellular carcinoma. We intend to conduct this study exploring the relation between BED_10_, efficacy and adverse reaction.

Our previous retrospective study [10] showed that the 1-, 2- and 3-year PFS, OS and distant metastasis- free survival (DMFS) rates were significantly higher in the BED_10_ ≥ 100Gy group than in the BED_10_ < 100Gy group (OS: *p* = 0.020; PFS: *p* = 0.017; DMFS: *p* = 0.012). However, LC in two groups were not of statistically significance. We speculated the result was relative to immune microenvironment. Previous studies [11, 12] reported the results of animal experiments of other types of carcinoma, which showed antitumor immunity significantly contributes to the superior response induced by higher single dose when the total dose remained the same. On the one hand, we conduct an animal experiment to explore antitumor immunity of hepatocellular carcinoma, and further experiments are in progress. On the other hand, our previous study was retrospective study, which may be biased. The result of this prospective study will be more credible.

Adverse reactions of hepatocellular carcinoma CK-SBRT are not ignored. Child-Pugh score and ICG R15 are all parameters to evaluate liver function, which are predicting factor of RILD in previous studies [13, 14]. It is worth mentioning that Child-Pugh score progression (increasing by two or more scores) is also a clinical metric for RILD [15]. As the BED_10_ of prescribed doses increase, so do the doses of the normal liver. Therefore, the study also focuses on predictors of RILD.

## Conclusion

Combining BED_10_ with radiation injury, the optimal CK-SBRT schedule of hepatocellular carcinoma is worth to explore.

## Data Availability

Materials and methods are available in the clinicaltrail.gov.

## Abbreviations

BED: Biologically effective dose
CK: CyberKnife
CP: Child-Pugh
CT: Computed tomography
ECOG: Eastern cooperative oncology group
GTV: Gross target volume
LC: Local control
MRI: Magnetic resonance imaging
OS: Overall survival
PD: Progressive disease
PTV: Planning target volume
RFA: Radiofrequency ablation
RILD: Radiation-induced liver disease
SBRT: Stereotactic body radiotherapy
